# How equitable is utilization of maternal health services in Uganda? Implications for achieving universal health coverage

**DOI:** 10.1186/s12913-023-09749-1

**Published:** 2023-07-26

**Authors:** Phiona Atuhaire, Elizabeth Kiracho-Ekirapa, John Mutenyo

**Affiliations:** 1 School of Economics, College of Business and Management Sciences, Makerere University, Kampala, Uganda; 2 School of Public Health, College of Health Sciences, Makerere University, Kampala, Uganda

**Keywords:** Maternal health, Socioeconomic groups, Inequalities, Universal Health Coverage

## Abstract

**Background:**

Maternal and neonatal mortality in Uganda remain persistently high. While utilisation of maternal health services has been shown to reduce the risk of maternal death, little is known about the inequalities in utilisation of maternal health services in Uganda. This study examined the inequalities in utilisation of maternal health services between 2006 and 2016 to draw implications for achieving universal health coverage.

**Methods:**

We used the Uganda Demographic Health Survey 2006, 2011 and 2016 to analyse inequalities in utilisation of antenatal care (ANC4+), skilled birth attendance (SBA), postnatal care (PNC) and a package of maternal health services. Equity ratios, concentration curves, concentration indices and regression analysis were used in the estimations.

**Results:**

Inequalities in utilization of single and a package of maternal health services reduced between 2005 and 2016, but remained pro-rich. Inequalities in utilisation of package of maternal health services were greater than for a single service. Women from the richest quintile were 4 times more likely to receive a package of care compared to the poorest women, but were just 1.5 times more likely to receive ANC4 + than those in the poorest quintile. In 2006 women in urban areas were 2.6 times more likely to receive a package of all three maternal health services than their rural counterpart and they had a relative advantage of 23.4% to utilize skilled birth delivery than the poorest women. Each additional year of schooling and living in urban areas was associated with 1.2 and 1.6% point increase in utilisation of a package of care respectively. Wealth, education and living in urban areas were positively associated with utilisation of all maternal healthcare.

**Conclusion:**

Declining inequalities in utilisation of maternal healthcare reflect a move towards achieving universal health coverage in Uganda. Pro-rich, education and urban-biased inequalities, imply the need for targeted interventions for the poor, less educated and rural women. Targeted voucher schemes, free distribution of birth kits for poorer and rural women, community-level mobilization to improve uptake of postnatal care, and promoting women’s education and incomes are feasible interventions to improve utilisation of maternal health services and equity.

**Supplementary Information:**

The online version contains supplementary material available at 10.1186/s12913-023-09749-1.

## Introduction

Uganda remains one of the high-burden countries with high absolute numbers of maternal deaths [1] in sub-Saharan Africa [2]. Skilled birth attendance has improved to 74% in 2016 from 42% to 2006. However, there remain rural-urban gaps, with 70% of women in rural areas accessing skill-births compared to 88% for urban women. During pregnancy, urban women were twice as likely to see a doctor (17%) than rural women (8%) [3]. Similarly, the rate of stillbirths and neonatal mortality equally remain high [4]. While Uganda’s maternal mortality ratio (MMR) declined from 687 to 343 per 100,000 live births between 1990 and 2015 [5–7], this is far from the Sustainable Development Goals (SDG) target of reducing the MMR to 70 per 100,000 live births. It also lags behind the Health Sector Development Plan (HSDP) national targets of 211 per 100,000 live births [7]. More recent statistics show that, despite the prioritization of maternal health services during the HSDP I, there was only a 4% point increase in the proportion of pregnant women who had four or more antenatal care visits (ANC4+) from 38% to 2015/16 to 42% in 2019/20, which remains short of the HSDP target of 47.5% [7]. Over the HSDP period, facility-based deliveries improved only slightly from 55% to 2015/16 to 59% in 2019/20 against a target of 89%, while Neonatal Mortality rate (NMR) has stagnated at 27/1000 live births against a target of 10/1000 live births [7].

Antenatal care (ANC) and skilled birth attendance are believed to prevent maternal and perinatal deaths [8, 9] and their coverage is routinely used to monitor progress towards improving maternal and neonatal health outcomes. Strategies to reduce the burden of maternal mortality in developing countries have proved to be among the most successful efforts to address a specific cluster of death causes [8]. These strategies included effective intrapartum-care, backed up by access to referral-level facilities. Some developing countries such as Indonesia, Guatemala, and Brazil reduced the risk of maternal death by 90–99%. Previous MMR of 1000 per 100,000 livebirths or greater risk of maternal mortality seen in some of these countries, has been reduced to as low as 10 per 100 000. Evidence has shown that in situations of high maternal mortality, opportunities for pregnant women to access health services such as antenatal care, facility based delivery and management of risk factors at child birth (haemorrhage or obstructed labor) are beneficial [9].

Previous studies in Uganda have explored factors associated with utilization of maternal health services. Coverage with any ANC over the last two decades has remained high at over 90%, less than 50% of the women went for ANC4 + visits. While 60% of the women had ANC4 + visits, in 2016 only 29% had ANC visits in the first trimester [6]. Women with higher levels of education were more likely to access early ANC, health facility delivery and early PNC [10–12]. For example, women with post-secondary education had a 33% chance of using professional childbirth care than the non-educated [12]. Similarly, being in higher wealth status as well as residing in urban area was associated with improved utilization of early PNC [11]. In Ethiopia, mothers with secondary or higher level of education, residing in urban area and high wealth status and working women had higher odds of delivering at health facilities [13]. These previous studies underscore the importance of assessing the utilization of maternal health services across socio-economic groups to understand the nature and trend of inequalities in a single and a continuum of care.

To achieve Universal Health Coverage (UHC), it is important that access to and utilisation of maternal health services improves in an equitable manner. Previous studies on Uganda examined the effect of socioeconomic and demographic factors on use of maternal healthcare, but did not explore the nature and trend of inequalities in utilisation of maternal health services [6, 11, 14, 15]. Understanding the nature and extent of inequities in utilisation of maternal health services over time is important in designing interventions to move towards UHC, including promoting those targeting particular groups. This study examined the nature and trend of inequalities in utilisation of at least ANC4 + visits, quality ANC, skilled- birth attendance (SBA), post-natal care (PNC) and a continuum of maternal health services across socio-economic groups and between rural and urban women.

## Methods

We used Uganda Demographic and Health Survey (UDHS) data for 2006, 2011 and 2016 collected by the Uganda Bureau of Statistics (UBOS) [16, 17, 3]. We used secondary data from UDHS module for women aged 15–49 years who had a birth in the last 5 years to estimate the socioeconomic inequalities using wealth, education and residence as socioeconomic indicators. The UDHS 2006 included a representative sample of 9,864 households selected in two stages. In the first stage, 321 clusters were selected from among a list of clusters sampled in the 2005–2006 Uganda National Household Survey [16]. In the second stage, households in each cluster were selected based on a complete listing of households. In the UDHS 2011, a representative sample of 10,086 households was selected in two stages. In the first stage, 404 EAs were selected from among a list of clusters sampled in the 2009/10 Uganda National Household Survey (2010 UNHS). In the second stage, households in each cluster were selected based on a complete listing of households. The UDHS 2016 used the sampling frame of the Uganda National Population and Housing Census (NPHC), conducted in 2014. A representative sample of 20,880 households (30 per EA or EA segment) was randomly selected.

One of the modules in the UDHS questionnaire on all women aged 15– 49 years of age who had had a birth in the last 5 years collects data on fertility and family planning, maternal and child health as well as details on antenatal and delivery care and other socio-demographic characteristics. Mothers are asked about utilization of maternal health services during the pregnancy for their most recent live birth in the 5 years preceding the survey. The total sample of women aged 15–49 years covered in UDHS 2006, 2011, and 2016 was 8,531, 9,247, and 18,506 respectively [16, 17, 3]. All women aged 15–49 who were either permanent residents of the selected households or visitors who stayed in the household the night before the survey were eligible to be interviewed. Details about the sampling procedure for these UDHS studies are reported under respective reports [16, 17, 3].

Inequalities in utilisation of maternal health services are estimated using the theoretical, analytical and methodological approaches for estimating inequities in health and healthcare as proposed by [18, 19] and as applied in many previous studies on maternal health [10, 11, 20]; [13]; [6], [12, 21]. The equity analysis in this paper is based on the egalitarian theory of justice as proposed by [22], which is the most used in empirical analysis of inequities. Egalitarian theory provides that all social groups are in equal need of a desired healthcare intervention and any distribution which deviates from this principle is considered to be inequitable. Based on the egalitarian theory, each pregnant woman deserves to access the required optimal ANC4 + visits and to give birth from a formal healthcare facility, assisted by a skilled health worker. Similarly she deserves to use postnatal care during the six weeks after birth [23].

We estimate equity ratios, concentration curves, the corresponding concentration indices and regression analysis to examine the nature and extent of socio-economic inequalities in use of a single and a continuum of maternal health services.

The equity ratio in use of a health service (e.g. ANC) is expressed as the percentage of the women in the top quintile that used the health service over the percentage of the women in the lowest quintile. The equity ratio ranges between 1 and infinity where lower values represent the lower levels of inequality. The concentration curve is a plot of the cumulative percentage of the sample ranked by SES (wealth quintile and years of schooling of the woman), and the corresponding cumulative proportion of Utilisation of the maternal health services (e.g. ANC4+, assisted births). Deviations from the diagonal (line of perfect equality) represent the extent of the inequalities. The concentration index is measured by the divergence of the concentration curve from the line of perfect equality [24]. The greater the absolute value of the index, the greater the extent of inequality and positive values reflect pro-rich inequalities – utilisation is concentrated among the women in top socioeconomic groups. The estimation model in the regression analysis includes a measure of socioeconomic status (Y); a vector for socio-demographic characteristics such as location, occupation of woman and the partner, age at last birth, distance to health facility, gestation age at first ANC visit and birth order, which are regressed upon the healthcare measure (HC) as the dependent variable (‘i.e.’ ANC4+, skilled birth attendance (SBA), Postnatal care (PNC) and a combination of maternal health services: ANC4+ &SBA; ANC4+ & SBA, &PNC). The dependent variable is binary (use/non-use), and we therefore run a logistic regression to obtain marginal/average effects of the explanatory variables on the dependent variable. We used p-values to examine the level of statistical significance of the estimated indices. The nature and extent of inequality is dependent on the magnitude and sign of the estimated concentration index and the p-value at 10%, 5%, and 1% level of confidence.

In addition to focusing on use of at least one ANC visit and ANC4+, we also analysed inequalities in quality ANC. Since utilization of ANC is already high (96% for any ANC and 58% for ANC4+) [3], it is important from a public health perspective to examine the quality of ANC pregnant women receive. An index for quality ANC was developed based on 8 parameters as recommended by the WHO which comprise the components of ANC visits. These include: a woman weighed, blood pressure measured, urine sample taken, blood sample taken, told about pregnancy complications, given iron supplements, given drugs for intestinal parasites, given SP for malaria prophylaxis. We generated a weighted index for quality ANC using multiple correspondent Analysis (MCA) which represents variations in quality of ANC received. We then generated 3 categories of quality ANC (low, medium and high) using the xtile (var), qn [3] syntax in STATA. The inequalities were then assessed for utilisation of high quality ANC. The choice of generating a weighted index was useful since in some years (2011 and 2016) and for the wealth quintiles-there were no women who obtained the eight components of ANC. Therefore, due to missing values, it was not feasible to use a simple summation of the components received.

## Results

### Socio-demographic characteristics of the sample

Table 1 presents summary statistics of the key socio-demographic characteristics of the sample. Most women had low levels of education with an average years of schooling of 4.4 in 2006 which slightly increased to 6.3 in 2016. Their partners were however better educated with 8 years of schooling on average. Most women and their partners were engaged in agricultural production, which is consistent with the structure of the Ugandan economy where over 70% of the population is involved in agriculture. Most women lived in rural areas with only 13% in urban areas in 2006 increasing to 23% in 2016.

**Table 1:** Socio-demographic characteristics

Characteristic	2006	2011	2016
Woman age at last birth (mean)	28.7	28.76	26.61
Woman years of schooling (mean)	4.413	5.365	6.303
Partner years of schooling (mean)	8.003	7.611	8.109
Household size (mean)	6.571	6.362	6.002
Woman occupation: Professional/technical/managerial	2.8%	4.3%	8.2%
Woman occupation: Sales and services	11.3%	19.9%	14.3%
Woman occupation: Agricultural/fishery	72.0%	55.5%	43.6%
Woman occupation: (Un)Skilled manual	5.0%		15.2%
Woman occupation: No work or household chores	8.9%	20.3%	18.7%
Partner occupation: Professional/technical/managerial	7.6%	7.2%	12.3%
Partner occupation: Sales and services	13.2%	23.3%	10.2%
Partner occupation: Agricultural/fishery	55.7%	65.2%	47.0%
Partner occupation: (Un)Skilled manual	18.2%		27.5%
Partner occupation: No work or household chores	5.3%	4.3%	2.9%
Wealth index: Lowest	21.3%	21.3%	20.9%
Wealth index: Second	21.7%	20.7%	20.4%
Wealth index: Middle	19.6%	19.4%	18.9%
Wealth index: Fourth	19.0%	18.0%	18.3%
Wealth index: Highest	18.3%	20.7%	21.5%
Household possesses radio/TV: Yes	61.0%	66.6%	59.6%
Residence: Urban	13.2%	16.1%	23.1%
Number of Observations	8531	9247	18,506

### Utilisation of maternal health services

The level of utilisation of maternal health services varied depending on the type of service. While about 98% of the women accessed at least one ANC visit, just 60% had received four or more visits in 2016 as shown in Table 2. Majority of women were starting ANC visits fairly late with only 29% starting within their first gestation period in 2016 compared to 16.5% in 2006. Utilisation of a single MHS (i.e. ANC4+, skilled birth attendance, or PNC) was higher than for a continuum of care. (‘i.e.’ 2 or all 3 maternal health services).

**Table 2:** Utilisation of maternal health services

Characteristic	2006	2011	2016
ANC visits: 4 + visits	47.2%	47.5%	59.9%
ANC visits: 1–3 visits	48.1%	48.0%	38.1%
ANC visits: 0 visits	4.6%	4.5%	2.0%
Quality ANC	33.2%	27.9%	29.2%
Gestation age at first visit: ≤ 3 months	16.5%	20.7%	29.1%
Gestation age at first visit: 4–6 months	62.5%	62.3%	61.6%
Gestation age at first visit: 7 + months	16.4%	12.5%	7.3%
Skilled delivery (SBA)	45.2%	60.4%	76.2%
Postnatal care: Yes	20.2%	37.7%	54.0%
4 ANC visits + SBA	26.7%	32.8%	49.0%
4 ANC visits + SBA + PNC	12.2%	18.9%	33.6%
Number of Observations	8531	9247	18,506

**Source**: Estimations from UDHS, 2006, 2011 & 2016 datasets

### Inequalities in utilisation of maternal health services

The inequalities in utilisation of maternal health services are estimated based on the equity ratio, concentration index (and corresponding concentration curve) and a multiple regression-based analysis. The estimated equity ratios for utilisation of ANC4+, SBA, a continuum of maternal health services and quality ANC are shown in Table 3. These ratios are derived from the estimated percentage utilisation of maternal health services by the top-most quintile versus the bottom quintile. The equity ratio shows the number of times the richest group is better off in terms of accessing care compared to the poorest group.

**Table 3:** Estimated equity ratios for use of ANC, SBA and a package of care, by Wealth Quintiles

Equity ratio (Richest/Poorest)	2006	2011	2016
ANC4 + visits	1.451	1.376	1.234
Quality ANC	1.436	1.555	1.772
SBA	2.625	1.987	1.437
PNC	2.762	2.030	1.474
ANC4 + visits + SBA	3.344	2.571	1.602
ANC4 + visits + SBA + PNC	4.333	3.176	1.824

**Source**: Computations based on UDHS data, 2006, 2011 and 2016

The higher the equity ratio the higher the inequality is against the poor (also referred to as pro-rich inequality). From Table 3 the nature of inequality in utilisation of maternal health services (both single and a continuum of services) is pro-rich. For example in 2016, women in the top most quintile were 1.8 times more likely to use a package of all three maternal health services than their counterparts in the poorest quintile. This implies that the poor women are disadvantaged regarding utilisation of maternal health services. It should be noted that the level of inequality has been reducing since 2006.

### Inequalities in utilisation of maternal health services by Residence

Inequalities were also assessed based on the place of residence to show the rural/urban differences in accessing maternal health services. The estimates in Table 4 shows that utilisation of maternal health services was relatively better for women in the urban compared to rural areas for all types of services. The inequality ratios estimated for each type of services shows by how much women in urban areas are more likely to access care than their rural counterparts. For example, in 2006, women in urban areas were 2.6 times more likely to receive a combination of all three maternal health services (ANC4 + SBA + PNC) than their rural counterparts. This reflects inequalities against the rural women. However, there has been a declining trend in the extent of inequalities (differences) in utilisation of maternal health services since 2006. This is a positive result in view of the national and global goal of moving towards universal health coverage in maternal health and across all healthcare services more generally.

**Table 4:** Utilisation of maternal health services by Residence

Estimate	2006	2011	2016
4 ANC visits: Rural	45.3%	45.7%	58.3%
4 ANC visits: Urban	59.7%	57.0%	65.2%
Inequality (Urban/Rural)	1.318	1.247	1.118
Quality ANC: Rural	28.8%	20.0%	25.3%
Quality ANC: Urban	58.7%	42.8%	45.8%
Inequality (Urban/Rural)	2.038	2.140	1.810
SBA: Rural	39.7%	55.0%	71.9%
SBA: Urban	83.3%	89.7%	90.6%
Inequality (Urban/Rural)	2.098	1.631	1.26
PNC: Rural	17.5%	33.4%	49.9%
PNC: Urban	39.9%	60.2%	67.4%
Inequality (Urban/Rural)	2.28	1.802	1.351
4 ANC visits + SBA: Rural	22.8%	29.0%	45.6%
4 ANC visits + SBA: Urban	52.4%	53.0%	60.0%
Inequality (Urban/Rural)	2.298	1.828	1.316
4 ANC visits + SBA + PNC: Rural	10.2%	15.4%	30.4%
4 ANC visits + SBA + PNC: Urban	26.1%	36.9%	44.5%
Inequality (Urban/Rural)	2.559	2.396	1.464

**Source**: Computations based on UDHS data, 2006, 2011 and 2016

### Inequality estimates based on concentration index and curves

**Fig. 1:**
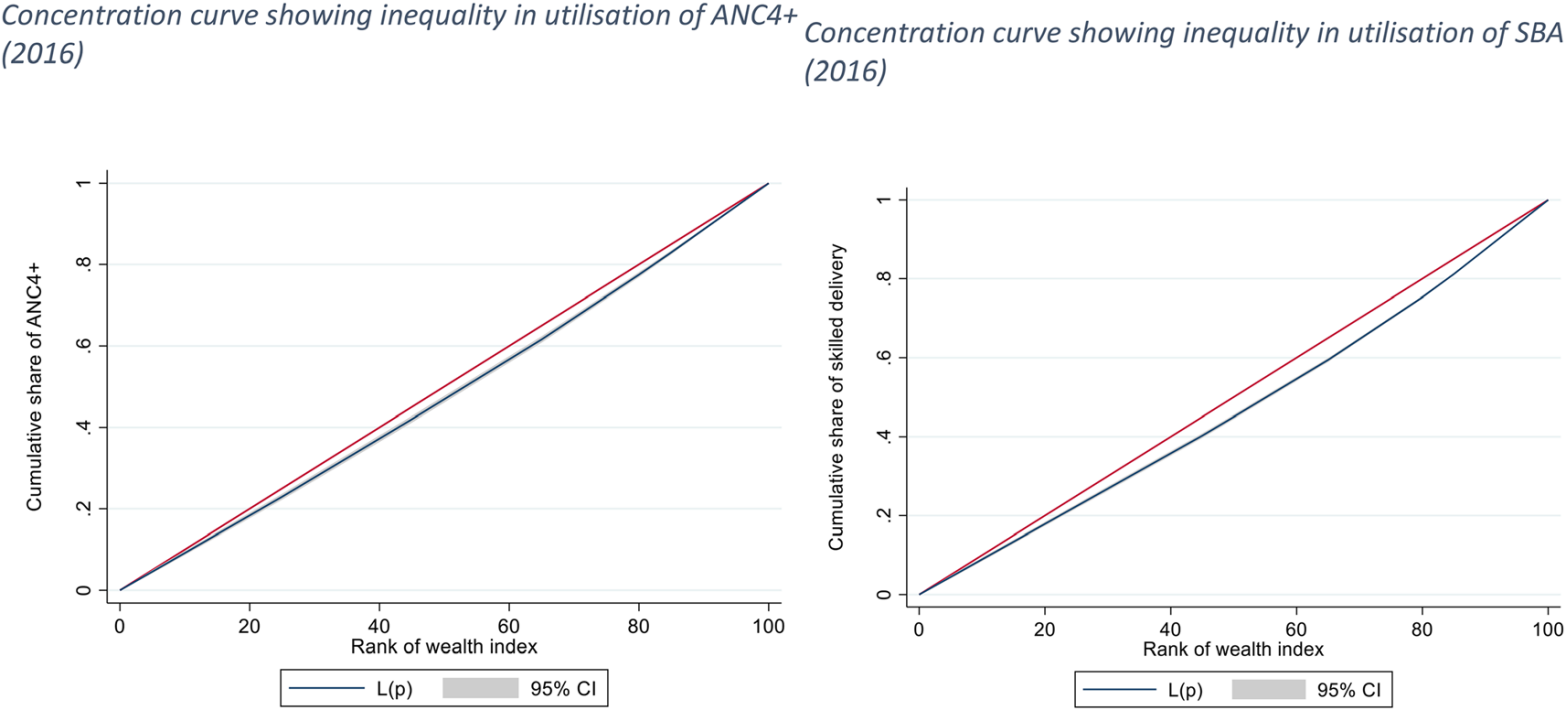
Concentration curves showing inequalities in utilisation of ANC4 + and SBA (2016)

**Fig. 2:**
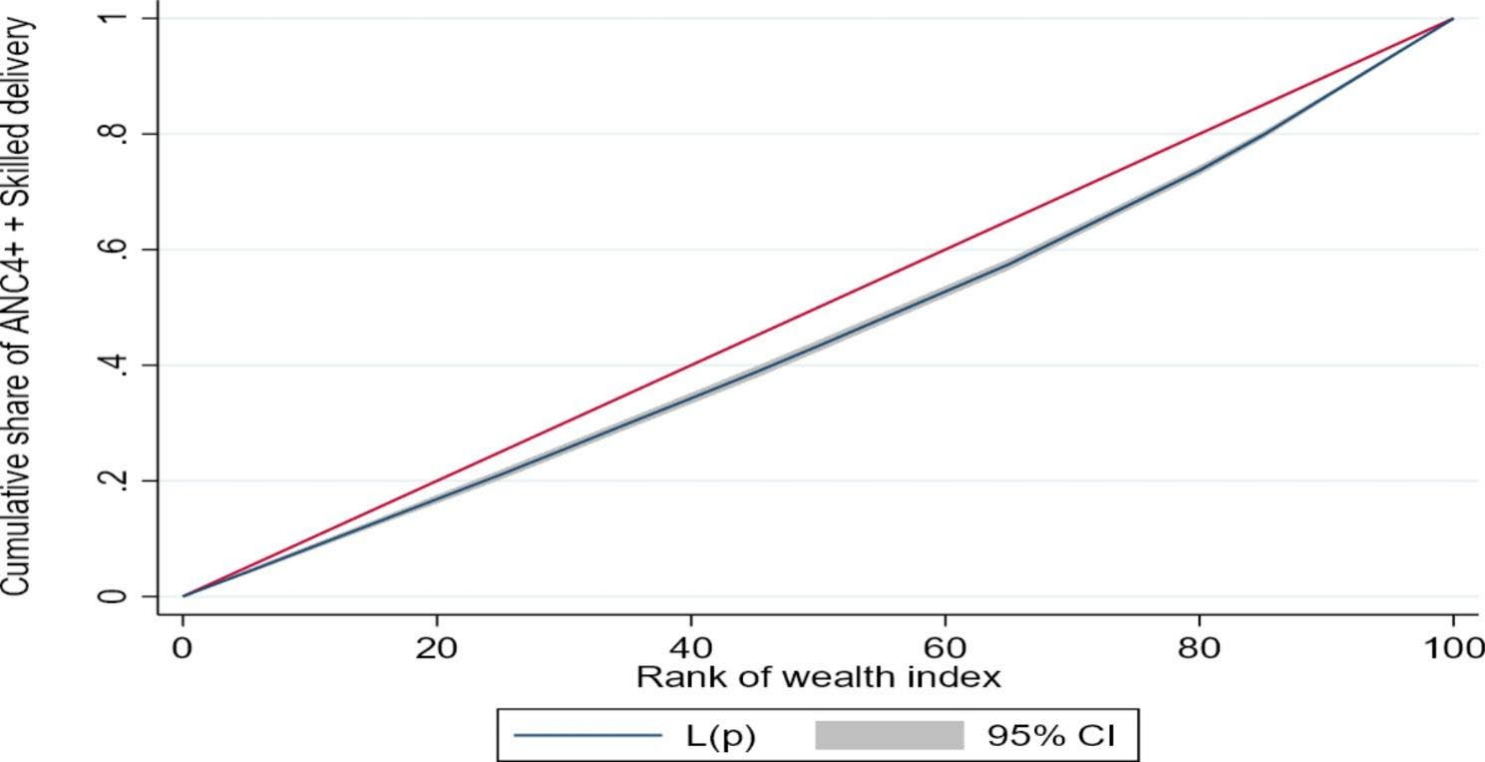
Concentration curve showing inequalities in utilisation of ANC4+, SBA and PNC Combined (2016)

**Fig. 3:**
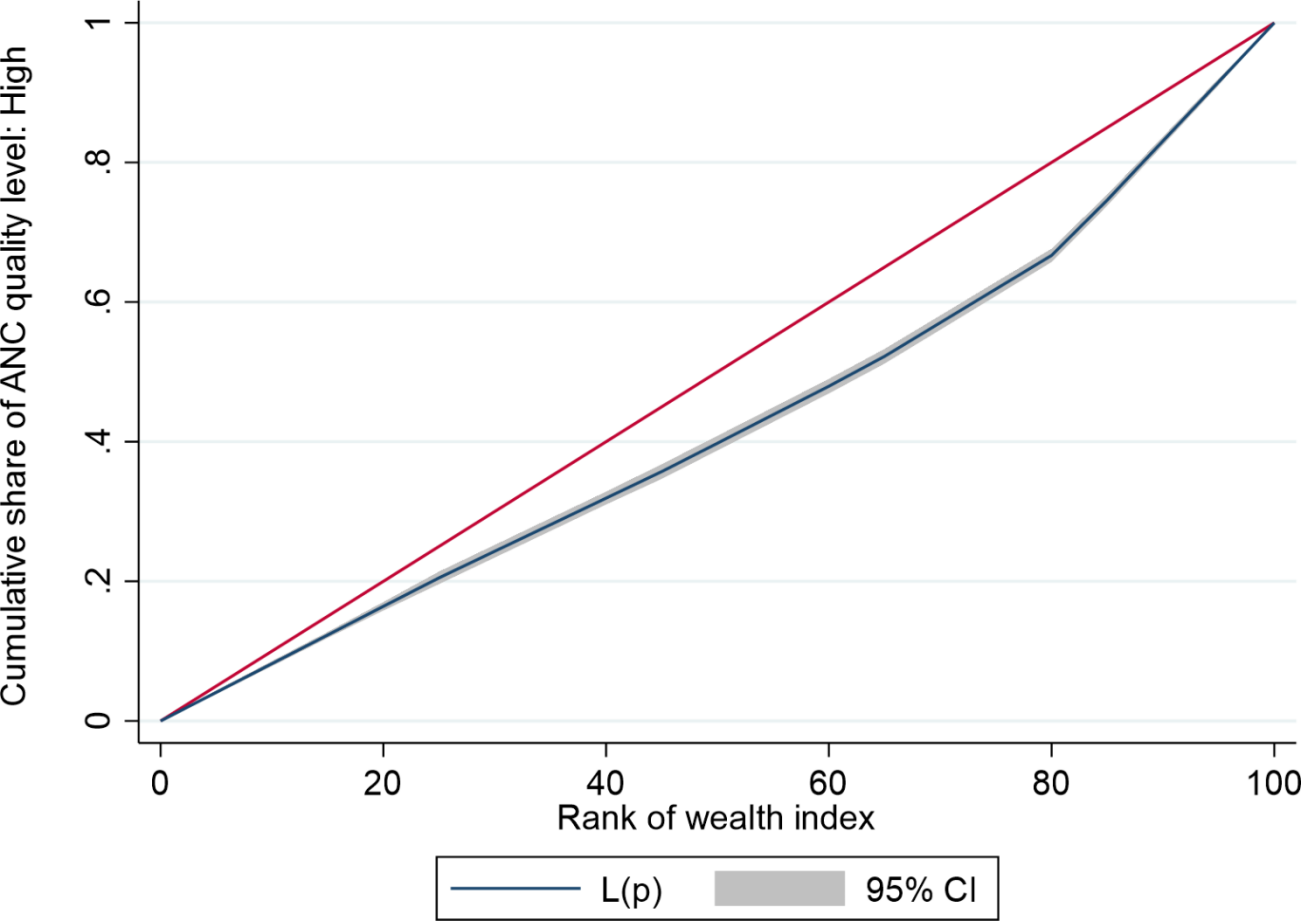
Concentration curve for inequalities in quality ANC 2006

**Fig. 4:**
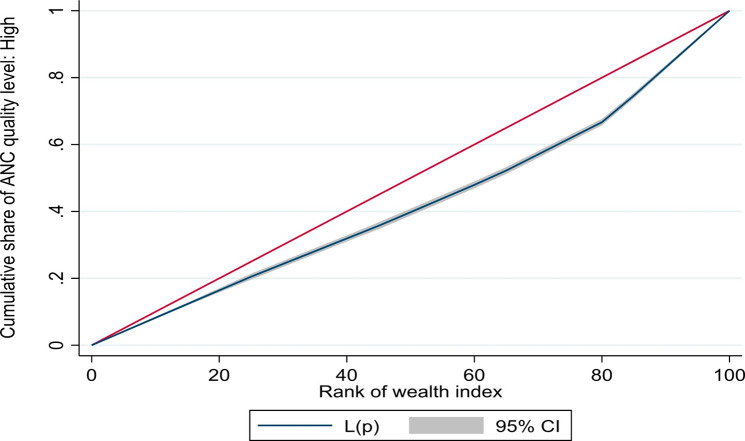
Concentration curve for inequalities in quality ANC 2011

**Fig. 5:**
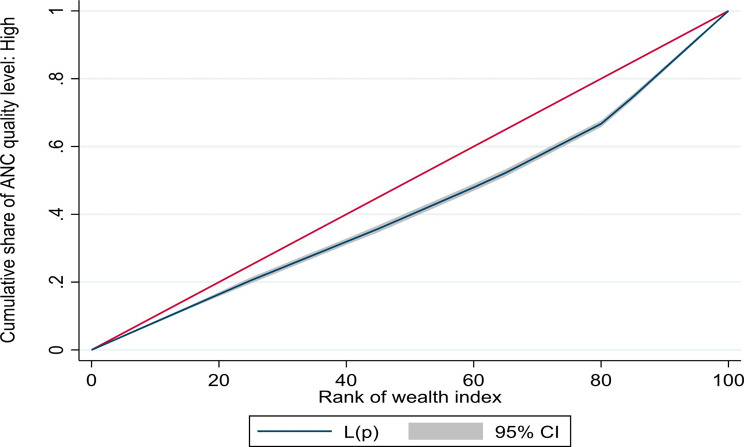
Concentration curve for Inequalities in Quality ANC 2016

We estimated the concentration index (and corresponding concentration curves) to counteract the limitations of the equity ratio which does not consider the distribution across all SEG. The concentration curves (based on UDHS 2016 data) for ANC4 + and SBA are shown in Fig. 1 below, while those for a continuum of maternal health services are given in Fig. 2. The concentration curves for quality ANC for the years, 2006, 2011, and 2016 are shown in Figs. 3, 4 and 5 respectively. Since the variable of interest is a health good (utilisation of maternal health services), the concentration curve lying below the line of perfect equality (the diagonal) implies inequality against the poor. The extent of divergence from the line of perfect equality shows the extent of the existing inequalities.

The inequalities in utilisation of SBA were larger than for ANC4+. Likewise, the inequalities in utilisation of a combination of ANC4+, SBA and PNC were larger than for a single maternal health service (Fig. 2). The inequalities in utilisation of Quality ANC slightly reduced between 2006 and 2016, but remained pro-rich (Figs. 3 and 4, and 5). The nature of inequalities in maternal health services based on the concentration curves are consistent with the estimated equity ratios.

The inequality estimate based on concentration indices are shown in Table 5 below. The positive sign shows that the inequalities are pro-rich (or against the poor).

**Table 5:** Concentration Indices for ACN4+, SBA, PNC and combination of care

	2006	2011	2016
ANC4: 4 + visits	0.068(0.010)***	0.068(0.011)***	0.042(0.007)***
Skilled delivery:	0.194(0.012)***	0.132(0.009)***	0.077(0.005)***
Postnatal care:	0.217(0.021)***	0.147(0.015)***	0.085(0.008)***
ANC4 + visits + SBA:	0.243(0.017)***	0.185(0.014)***	0.096(0.008)***
ANC4 + visits + SBA + PNC:	0.304(0.027)***	0.251(0.023)***	0.130(0.011)***

Standard errors in parentheses ^*^p < 0.05, ^**^p < 0.01, ^***^p < 0.001

Inequalities in a single maternal health service and a continuum of care have been reducing over the period 2006 to 2016 as shown by the decline in the magnitude of the concentration indices over this same period but remain pro-rich. Relative to utilisation of a single package of maternal health services (e.g. ANC4 + or SBA), inequalities are greater in accessing a combination of services (“i.e.” ANC4 + SBA + PNC, & ANC4 + SBA). Inequalities are greater for jointly accessing all three maternal health services (ANC4 + SBA + PNC) than just two of the full packages of comprehensive maternal healthcare (i.e. ANC4 + SBA). The results of the concentration indices, which consider the distribution across all population groups are consistent with the estimated equity ratios, which also depicted a reduction in the level of inequality overtime.

### Relationship between SES and utilisation of maternal health services: a multiple regression analysis

This section presents findings on the predictors of utilisation of quality ANC, skilled birth attendance, PNC, and a continuum of health care. The SES variables of interest in this analysis were- place of residence, wealth status and education of the mother. The socioeconomic inequalities in use of quality ANC are shown in Table 6. The magnitude and nature of inequality are reflected by the size and the sign of the estimated coefficient respectively. The estimates are margins which show the percentage changes in the dependent variable due to a percentage change in the SES indicator. The results reported in Tables 6 to 8 are for the SES variables of interest (‘i.e.’ wealth, education of a woman and residence). The richest quintile has been taken as the base to calculate the marginal effects in the regression analysis. Detailed results including confounders are shown in the supplementary file.

Education was positively associated with utilisation of quality ANC over the time period. An additional year of schooling was associated with a 2.5% point increase in utilisation of quality ANC in 2006 and 1.6% point increase by 2016. Wealthier women were more likely to receive quality ANC compared to those in the lower wealth quintiles. In 2006, women in the lowest wealth quintile were 21.3% points less likely to receive utilisation of quality ANC compared to those in the top most quintile slightly reducing to 20.0% points by 2016. In 2006, women residing in urban areas were 38.7% points more likely to access quality ANC services compared to the rural areas. However this relative advantage reduced to just 8.1% points by 2016 which shows significant decline in inequalities.

**Table 6:** Socio-economic inequalities in use of ANC4 + and quality ANC

		ANC4+			Quality ANC		
	2006	2011	2016	2006	2011	2016	
Woman years of schooling	0.009 (0.002)^***^	0.001 (0.003)	0.005 (0.002)^**^	0.025 (0.006)^***^	0.029 (0.005)^***^	0.016 (0.003)^***^	
Wealth index: Lowest	-0.062 (0.033)	-0.111 (0.041)^**^	-0.066 (0.024)^**^	-0.213 (0.071)^***^	-0.026 (0.068)	-0.200 (0.052)^***^	
Wealth index: Second	-0.087 (0.034)^*^	-0.104 (0.034)^**^	-0.045 (0.023)	-0.220 (0.064)^***^	-0.163 (0.057)^***^	-0.246 (0.050)^***^	
Wealth index: Middle	-0.102 (0.031)^***^	-0.089 (0.033)^**^	-0.042 (0.021)^*^	-0.231 (0.066)^***^	-0.206 (0.055)^***^	-0.219 (0.043)^***^	
Wealth index: Fourth	-0.107 (0.030)^***^	-0.037 (0.033)	-0.010 (0.021)	-0.177 (0.059)^***^	-0.144 (0.050)^***^	-0.143 (0.038)^***^	
Residence: Urban	-0.013 (0.031)	-0.017 (0.029)	0.012 (0.017)	0.387 (0.075)^***^	0.277 (0.074)^***^	0.081 (0.038)^**^	
R squared	0.195	0.176	0.167	0.221	0.303	0.296	
Observations	4947	4868	10,263	4947	4868	10,263	

Standard errors in parentheses ^*^p < 0.10, ^**^p < 0.05, ^***^p < 0.01

### Inequalities in skilled birth delivery

Results for the estimated inequalities in utilisation of SBA (Table 7) show that a unit increase in the years of school for the mother increased the likelihood of accessing SBA by 1.9%, 1.5%, and 1.1% points. Likewise, in 2006 women in the lowest wealth quintile were 15.1% points less likely to be delivered by a SBA compared to women in the richest SEG. The relative advantage of the women in the richest SEG to access skilled birth delivery increased to 23.4% points in 2011 before reducing to 10.8% points, but the distribution remained inequitable against the poor. The relative advantage between women in the richest group and those in the 4th, 3rd and 2nd was correspondingly lower, and declined between 2006 and 2016. That is to say, the advantage of the women in the richest group to access SBA relative to those in the 4th SEGs is lower than that relative to the women in the 3rd quintile, and so on. In 2006, women in urban areas were 13.5% points more likely to have a birth assisted by a skilled health worker compared to those in rural areas. This relative advantage reduced to 9.4% points in 2011 and further to 4.8% points in 2016, depicting a declining trend in inequalities. Estimates for the full regression model are in the supplementary file.

**Table 7:** Socio-economic inequalities in skilled birth delivery and postnatal care

		SBA			PNC		
	2006	2011	2016	2006	2011	2016	
Woman years of schooling	0.019 (0.003)^***^	0.015 (0.003)^***^	0.011 (0.002)^***^	0.011 (0.003)^***^	0.017 (0.003)^***^	0.014 (0.002)^***^	
Wealth index: Lowest	-0.151 (0.042)^***^	-0.234 (0.038)^***^	-0.108 (0.024)^***^	-0.049 (0.033)	-0.122 (0.044)^**^	-0.081 (0.030)^**^	
Wealth index: Second	-0.142 (0.037)^***^	-0.180 (0.031)^***^	-0.124 (0.019)^***^	-0.078 (0.031)^*^	-0.131 (0.040)^**^	-0.133 (0.028)^***^	
Wealth index: Middle	-0.138 (0.034)^***^	-0.166 (0.029)^***^	-0.078 (0.017)^***^	-0.087 (0.032)^**^	-0.157 (0.039)^***^	-0.115 (0.026)^***^	
Wealth index: Fourth	-0.072 (0.032)^*^	-0.136 (0.028)^***^	-0.040 (0.014)^**^	-0.073 (0.028)^**^	-0.119 (0.039)^**^	-0.088 (0.024)^***^	
Residence: Urban	0.135 (0.044)^**^	0.094 (0.025)^***^	0.048 (0.014)^***^	0.043 (0.030)	0.063 (0.035)	0.043 (0.022)	
R squared	0.206	0.157	0.120	0.121	0.107	0.075	
Observations	4947	4868	10,263	4947	4868	10,263	

Standard errors in parentheses ^*^p < 0.05, ^**^p < 0.01, ^***^p < 0.001

Inequalities in utilisation of postnatal care had an increasing trend against the poor between 2006 and 2016 (Table 7). More educated mothers were more likely to access PNC compared to their less educated counterparts. Whereas an additional year of schooling increased the likelihood of receiving PNC by 1.1% points, this increased to 1.7% points in 2011 and slightly reduced to 1.4% points in 2016 reflecting an increase in inequalities based on the education level of the woman. Compared to women in the top-most wealth quintile, women in the lower wealth quintiles were less likely to access postnatal care with an increasing trend in inequality over the 10 year period.

### Inequalities in utilisation of a package of maternal health services

The findings on the level and nature of inequalities in accessing a combination of maternal health services are reported in Table 8 ((ANC4 + and SBA) and (ANC4+, SBA and PNC)).

**Table 8:** Socioeconomic inequalities in utilisation of ANC4+& SBA and a continuum of care

		ANC + SBA			ANC + SBA + PNC	
	2006	2011	2016	2006	2011	2016
Woman years of schooling	0.013 (0.003)^***^	0.007 (0.003)^**^	0.010 (0.002)^***^	0.008 (0.002)^***^	0.010 (0.002)^***^	0.012 (0.002)^***^
Wealth index: Lowest	-0.164 (0.036)^***^	-0.200 (0.041)^***^	-0.094 (0.026)^***^	-0.067 (0.025)^***^	-0.135 (0.032)^***^	-0.116 (0.025)^***^
Wealth index: Second	-0.152 (0.034)^***^	-0.154 (0.034)^***^	-0.088 (0.024)^***^	-0.081 (0.024)^***^	-0.132 (0.032)^***^	-0.120 (0.022)^***^
Wealth index: Middle	-0.161 (0.031)^***^	-0.139 (0.033)^***^	-0.076 (0.022)^***^	-0.067 (0.024)^***^	-0.105 (0.029)^***^	-0.079 (0.024)^***^
Wealth index: Fourth	-0.137 (0.031)^***^	-0.108 (0.031)^***^	-0.039 (0.022)	0.018 (0.010)^*^	-0.007 (0.012)	0.001 (0.012)
Residence: Urban	0.054 (0.034)	0.035 (0.029)	0.027 (0.018)	-0.016 (0.010)^*^	-0.019 (0.012)	-0.023 (0.012)^**^
R squared	0.187	0.156	0.153	4947	4868	10,263
Observations	4947	4868	10,263			

Standard errors in parentheses ^*^p < 0.05, ^**^p < 0.01, ^***^p < 0.001

From the table, being more educated, in a higher wealth quintile as well as living in the urban area were positively associated with accessing a combination of ANC4 + and skilled birth attendance. This reflects inequalities against the women who were socioeconomically disadvantaged. However the inequalities declined between 2006 and 2016, reflecting a positive trend towards UHC. Unlike the inequalities in accessing ANC and SBA which declined over-time, those for a combination of all three services increased between 2006 and 2016. Women who were more educated, of a higher wealth status as well as living in the urban area were more likely to access a combination of all three maternal health services compared to their counterparts.

## Discussion

This paper set out to examine the nature and extent of inequalities in utilisation of at least four ANC visits (ANC4+), including quality ANC, skilled-birth attendance (SBA), post-natal care (PNC), and a continuum of services across socioeconomic groups and between rural and urban women overtime. We used the common analytical approaches for estimating inequities in health and healthcare as proposed by [18, 19] to estimate equity ratios, concentration indices and regression analysis based on UDHS data for 2006, 2011 and 2016. The methodological contribution of this paper were three fold: first to analyse the trend in inequalities at multiple cross-section over a 10 year period while the previous studies were looking at single data points. Secondly, we estimate utilisation and inequalities in a package of care by considering those women who had two (ANC and SBA) or all three (ANC + SBA + PNC) maternal health services. Thirdly we estimate inequalities in utilisation of quality ANC based on a weighted index of quality parameter as explained in the methods section. When high levels of utilisation of at least one ANC visit has been achieved, attention should be directed to the quality of ANC being provided. About 98% of the women received at least one ANC visit in 2016, which is obviously a high level of utilisation. Under such high levels of utilisation, the issue of quality become important. But less so at lower coverage rates, where any level of quality would still be beneficial.

The inequalities in utilisation of maternal health services have declined since 2006, but remain pro-rich. The decline in inequalities is a positive trend in relation to achieving equitable utilisation of maternal health services, which is a key factor in moving the country towards universal health coverage. However, wealthier, more educated and urban-based women have an advantage in using maternal health services compared to their poor, less educated and rural-based counterparts, who are also the majority in Uganda. These findings are similar to those by [6, 10–12, 14] studies on Uganda who found that a woman’s wealth status, years of schooling education and living in urban areas were positively associated with likelihood of using maternal health services. Empirical work by [14, 25–28] and a study on utilisation of maternal health services in five African countries by [29] reported similar findings. Our study adds value to the exiting knowledge by assessing the trends in inequalities, as well as considering utilisation of a package of MHS, which previous studies did not explore. Thus, making maternal health services accessible to poorer, less educated and rural-based women remains a priority in order to achieve UHC.

Utilisation of a package of all three maternal health services (ANC4+, SBA and PNC) remain quite low and significantly concentrated among the richer, more educated and urban based women. This is particularly so because of the very few women that access postnatal care after delivery. This could be because the cost of care is more affordable to such women, they have better access to health facilities in the urban areas, and have better access to information and knowledge about the benefits of maternal health services (MHS). A few studies have shown that better educated women have greater capacity to access and process available information on healthcare and take action to access care [14, 27–34]. They also tend to become more aware of the potential dangers of not accessing necessary care. Similarly, wealthier women have the capacity to meet the financial cost of accessing care, such as transport cost, which remain a barrier to healthcare access. The finding that more educated women with a higher socioeconomic status were more likely to receive maternal health services both within the rural and urban areas is consistent with those found in other similar settings in low-income countries [14, 25, 27, 29, 31, 35, 36]. This suggests that over the long-term, increasing education opportunities for girls and young women, and interventions to improve household incomes, has a direct effect on improving utilisation of maternal health services and other forms of healthcare generally. Increasing utilisation of care, and in an equitable manner are important for the attainment of UHC.

Utilisation of any ANC and ANC4 + services has improved since 2006. Despite this improvement women accessing quality ANC remain few and or are concentrated among the high income groups. This has implications on maternal health outcomes in the underserved socio-economic groups. Women who were more likely to access quality ANC were those from wealthier quintiles, more educated, and from urban areas. Thus interventions targeted at the poorer and rural based women are necessary to improve overall maternal health outcomes. The literature shows that receiving ANC services early on during the pregnancy, and making sufficient number of ANC visits (ANC4 + revised to ANC8 + by WHO since 2016) are important for better health outcomes during delivery and the postnatal period [37, 38]. However, the findings in this study showed that for some SEGs the proportion of women who delivered at a health facility (SBA) was greater than those who had received ANC4+. This suggests that some women although delivered at a health facility in the hands of a skilled birth attendant, they had not received the recommended number of ANC visits.

Similar to ANC, there has been a steady decline in the level of inequalities in utilisation of skill-birth deliveries overtime. Moreover, the proportion of the less educated and poorer women accessing skilled-birth deliveries has been increasing overtime, while the level of inequality has been declining. These findings reflect the positive effect of the interventions by government and other partners to improve utilisation of maternal health services across all population groups and have important implications for achieving UHC. The interventions include: government policy to have a functional Health Centre III with maternity services at sub-county level to increase utilisation of health facilities, improved staffing levels, and reducing stock-outs of essential medicines and health supplies at health facilities, among others. The urban/rural inequalities can be attributed to the imbalances in availability of sources of care between rural and urban areas. There are more healthcare providers for maternal health services (and other healthcare services) in the urban compared to rural areas. From the health system perspective, it will be important to increase sources of care for maternal health services in rural areas, through expanding the public healthcare infrastructure which provides maternal health services, as well as promoting Public-private partnerships initiatives for health care to expand services in areas where the public health system is not well-established to provide needed care. Social mobilisation of women through health campaigns at the community level within the Village Health Teams (VHT) system can help to ensure women start their ANC visits early during pregnancy.

Women who had no ANC visits were less likely to utilise skilled-birth delivery compared to those who had at least one ANC visit. This implies that a woman’s utilisation of ANC during pregnancy highly influences their utilisation of skilled birth delivery. This finding underscores the importance of providing an integrated package of maternal health services for all pregnant mothers. Previous studies have shown that a woman having four or more ANC visits and receiving better quality of ANC affected women’s subsequent use of SBA. Delivery at a health facility is significantly associated with the continuation from having SBA to receiving PNC- the completion of continuum of care [39]. The findings on the low level of utilisation of a continuum of care for the poorer women and those living in rural areas contributes to the global debates about promoting access to a continuum of maternal health care with particular emphasis on skilled birth attendance, which has been shown to be an important intervention to reduce maternal deaths [39]. This study has revealed declining trends in inequalities in utilisation of maternal health services, which is an important step towards achieving universal health coverage. It has also shown that inequalities, though declining, remain pro-rich and urban-biased, implying the need to improve utilisation among the poor women and those in rural areas. Inequalities in utilisation in continuum of care remain significantly larger than for a single maternal health care. Thus interventions to promote utilisation of a package of care from ANC visits to delivery at a health facility and post-natal care are important for further reducing the current levels of maternal mortality. Finally the paper has shown that while utilisation of ANC had improved, fewer women were obtaining good quality ANC as defined by the number of ANC components received overall during the antenatal care visits as recommended by WHO. The reasons for this observation were beyond the scope of this study, but should be explored in future research.

.

The limitation of this paper which merit discussion relates to using ANC4 + as an indicator of recommended ANC visits as opposed to the current policy of eight or more ANC visits. This however does not affect the finding of this study, since the latest UDHS 2016 used in the analysis was collected before the new policy was introduced. Thus, priory to the new policy, health workers as well as pregnant women were following recommendation of ANC4+. In view of the current WHO recommendation for ANC + 8 [40], it is quite likely that inequalities for ANC8 + would be much higher than what is estimated in this paper for ANC4+, since very few women would actually aim to go for twice or more times than the recommended minimum of four visits.

## Conclusion

Inequalities in utilisation of single and a continuum of maternal health services have declined since 2006, which is a positive trend for achieving UHC. Overall, utilisation of maternal health services improved between 2006 and 2016, including among women with lower socioeconomic status. The proportionate increase in the women in lower wealth quintiles receiving maternal health care could be attributed to the range of interventions implemented over the period which focused on improving access for all women, including the poor as discussed above. However, the pro-rich nature of existing inequalities suggests that there is need for targeted interventions to improve access among the poorer, less educated and rural women. Targeted interventions to improve utilisation of maternal health services for the poor could include voucher schemes to enable poorer women access care, especially for SBA and PNC where inequalities are larger than for ANC4+. Improving utilisation of these services will eventually address the existing inequalities in receiving a continuum of maternal health care.

The relatively high inequalities in utilisation of a continuum of care and quality ANC reflect the need for health sector and health practitioners working on improving maternal health outcomes to deliberately focus on interventions that target these two components of care. Continuous assessment of the trends in utilisation of maternal health services is important to highlight existing inequalities, which should be addressed such that no group of women are left behind. Providing quality and a continuum of maternal health care to all socioeconomic groups is important for reducing maternal deaths. An increase in utilisation of maternal health care through implementation of effective interventions, and for all groups provide an opportunity for the country to move towards achieving UHC. Government in collaboration with partners should implement targeted interventions such as voucher schemes to improve utilisation of maternal health services to enable poorer women access care, especially for SBA and PNC where inequalities are larger than for ANC4+, to move towards achieving universal health coverage.

## Electronic supplementary material

Below is the link to the electronic supplementary material.

**Supplementary Table 1**: Predictors in Utilisation of Quality Antenatal Care. **Supplementary Table 2**: Predictors of Utilisation of Skilled Birth Attendance. **Supplementary Table 3**: Factors associated with utilisation of Postnatal Care services. **Supplementary Table 4**: Predictors of utilisation of ANC4+ visits and Skilled Birth Attendance combined. **Supplementary Table 5**: Predictors of utilisation of ANC4+ visits, Skilled Birth Attendance and PNC combined

## Data Availability

The datasets analyzed during the current study are publicly available in the U.S. Agency for International Development repository, [PERSISTENT WEB LINK TO DATASETS]. https://dhsprogram.com/data/dataset/Uganda_Standard-DHS_2011.cfm?flag=0.
