# Supplementary material for: How equitable is utilization of maternal health services in Uganda? Implications for achieving universal health coverage
Source: BMC Health Serv Res. 2023 Jul 26;23:800. doi: 10.1186/s12913-023-09749-1 (PMC10369811; doi:10.1186/s12913-023-09749-1)
Supplement: Supplementary file 1 — Supplementary Table 1: Predictors in Utilisation of Quality Antenatal Care. Supplementary Table 2: Predictors of Utilisation of Skilled Birth Attendance. Supplementary Table 3: Factors associated with utilisation of Postnatal Care services. Supplementary Table 4: Predictors of utilisation of ANC4+ visits and Skilled Birth Attendance combined. Supplementary Table 5: Predictors of utilisation of ANC4+ visits, Skilled Birth Attendance and PNC combined [file 12913_2023_9749_MOESM1_ESM.docx]

**Detailed regression results for utilisation of a single and a package of maternal health services**

**Supplementary Table 1: Predictors in Utilisation of Quality Antenatal Care**

|  | 2006 | 2011 | 2016 |
| --- | --- | --- | --- |
| Woman age at last birth | 0.058 (0.019)^***^ | 0.037 (0.019)^*^ | 0.030 (0.014)^**^ |
| Woman age at last birth squared | -0.001 (0.000)^**^ | -0.000 (0.000) | -0.000 (0.000) |
| Woman years of schooling | 0.025 (0.006)^***^ | 0.029 (0.005)^***^ | 0.016 (0.003)^***^ |
| Partner years of schooling | 0.002 (0.001) | -0.003 (0.002) | 0.002 (0.001)^**^ |
| Woman occupation: Professional/technical/managerial | 0.434 (0.097)^***^ | 0.188 (0.075)^**^ | 0.182 (0.040)^***^ |
| Woman occupation: Sales and services | 0.133 (0.063)^**^ | 0.137 (0.041)^***^ | 0.228 (0.034)^***^ |
| Woman occupation: (Un)Skilled manual | 0.138 (0.068)^**^ |  | 0.153 (0.033)^***^ |
| Woman occupation: No work or household chores | 0.021 (0.068) | 0.072 (0.040)^*^ | 0.073 (0.043)^*^ |
| Partner occupation: Professional/technical/managerial | 0.000 (0.063) | 0.062 (0.059) | 0.123 (0.034)^***^ |
| Partner occupation: Sales and services | 0.078 (0.047)^*^ | 0.079 (0.036)^**^ | 0.038 (0.035) |
| Partner occupation: (Un)Skilled manual | 0.062 (0.047) |  | 0.076 (0.027)^***^ |
| Partner occupation: No work or household chores | 0.080 (0.069) | -0.152 (0.076)^**^ | 0.178 (0.055)^***^ |
| Wealth index: Lowest | -0.213 (0.071)^***^ | -0.026 (0.068) | -0.200 (0.052)^***^ |
| Wealth index: Second | -0.220 (0.064)^***^ | -0.163 (0.057)^***^ | -0.246 (0.050)^***^ |
| Wealth index: Middle | -0.231 (0.066)^***^ | -0.206 (0.055)^***^ | -0.219 (0.043)^***^ |
| Wealth index: Fourth | -0.177 (0.059)^***^ | -0.144 (0.050)^***^ | -0.143 (0.038)^***^ |
| Household possesses radio/TV: Yes | -0.033 (0.033) | -0.030 (0.038) | -0.049 (0.022)^**^ |
| Residence: Urban | 0.387 (0.075)^***^ | 0.277 (0.074)^***^ | 0.081 (0.038)^**^ |
| Distance is a problem: Yes | -0.060 (0.034)^*^ | -0.038 (0.027) | -0.046 (0.025)^*^ |
| Birth order: 3rd - 4th | -0.153 (0.050)^***^ | -0.120 (0.045)^***^ | -0.037 (0.028) |
| Birth order: 5th + | -0.225 (0.065)^***^ | -0.264 (0.056)^***^ | -0.132 (0.044)^***^ |
| Gestation age at first visit: 4 - 6 months | -0.050 (0.043) | -0.146 (0.034)^***^ | -0.084 (0.024)^***^ |
| Gestation age at first visit: 7+ months | -0.266 (0.052)^***^ | -0.405 (0.057)^***^ | -0.306 (0.052)^***^ |
| R squared | 0.221 | 0.303 | 0.296 |
| Observations | 4947 | 4868 | 10263 |

**Supplementary Table 2: Predictors of Utilisation of Skilled Birth Attendance**

|  | 2006 | 2011 | 2016 |
| --- | --- | --- | --- |
| Woman age at last birth | 0.014 (0.009) | -0.013 (0.010) | -0.002 (0.006) |
| Woman age at last birth squared | -0.000 (0.000) | 0.000 (0.000) | 0.000 (0.000) |
| Woman years of schooling | 0.019 (0.003)^***^ | 0.015 (0.003)^***^ | 0.011 (0.002)^***^ |
| Partner years of schooling | 0.000 (0.001) | 0.002 (0.001)^*^ | 0.001 (0.000) |
| Woman occupation: Professional/technical/managerial | 0.096 (0.048)^*^ | 0.049 (0.041) | 0.035 (0.017)^*^ |
| Woman occupation: Sales and services | 0.102 (0.027)^***^ | 0.094 (0.021)^***^ | 0.049 (0.016)^**^ |
| Woman occupation: (Un)Skilled manual | 0.059 (0.034) |  | 0.017 (0.016) |
| Woman occupation: No work or household chores | 0.129 (0.030)^***^ | 0.083 (0.023)^***^ | 0.045 (0.018)^*^ |
| Partner occupation: Professional/technical/managerial | 0.008 (0.031) | 0.040 (0.028) | 0.056 (0.013)^***^ |
| Partner occupation: Sales and services | 0.059 (0.022)^**^ | 0.038 (0.019)^*^ | 0.020 (0.016) |
| Partner occupation: (Un)Skilled manual | 0.064 (0.022)^**^ |  | 0.017 (0.011) |
| Partner occupation: No work or household chores | 0.050 (0.037) | -0.014 (0.038) | 0.003 (0.029) |
| Wealth index: Lowest | -0.151 (0.042)^***^ | -0.234 (0.038)^***^ | -0.108 (0.024)^***^ |
| Wealth index: Second | -0.142 (0.037)^***^ | -0.180 (0.031)^***^ | -0.124 (0.019)^***^ |
| Wealth index: Middle | -0.138 (0.034)^***^ | -0.166 (0.029)^***^ | -0.078 (0.017)^***^ |
| Wealth index: Fourth | -0.072 (0.032)^*^ | -0.136 (0.028)^***^ | -0.040 (0.014)^**^ |
| Household possesses radio/TV: Yes | 0.007 (0.019) | -0.034 (0.018) | 0.002 (0.012) |
| Residence: Urban | 0.135 (0.044)^**^ | 0.094 (0.025)^***^ | 0.048 (0.014)^***^ |
| Distance is a problem: Yes | -0.058 (0.014)^***^ | -0.003 (0.015) | -0.052 (0.012)^***^ |
| Birth order: 3rd - 4th | -0.064 (0.023)^**^ | -0.055 (0.024)^*^ | -0.050 (0.013)^***^ |
| Birth order: 5th + | -0.092 (0.029)^**^ | -0.051 (0.032) | -0.070 (0.018)^***^ |
| Gestation age at first visit: 4 - 6 months | -0.027 (0.020) | -0.049 (0.019)^*^ | -0.056 (0.009)^***^ |
| Gestation age at first visit: 7+ months | -0.085 (0.025)^***^ | -0.087 (0.030)^**^ | -0.165 (0.023)^***^ |
| ANC visits: 0 visits | -0.254 (0.031)^***^ | -0.270 (0.045)^***^ | -0.314 (0.040)^***^ |
| R squared | 0.206 | 0.157 | 0.120 |
| Observations | 4947 | 4868 | 10263 |

**Supplementary Table 3: Factors associated with utilisation of Postnatal Care services**

|  | 2006 | 2011 | 2016 |
| --- | --- | --- | --- |
| Woman age at last birth | 0.016 (0.007)^*^ | -0.004 (0.009) | 0.003 (0.007) |
| Woman age at last birth squared | -0.000 (0.000) | 0.000 (0.000) | -0.000 (0.000) |
| Woman years of schooling | 0.011 (0.003)^***^ | 0.017 (0.003)^***^ | 0.014 (0.002)^***^ |
| Partner years of schooling | 0.002 (0.001)^*^ | 0.000 (0.001) | 0.001 (0.001) |
| Woman occupation: Professional/technical/managerial | 0.225 (0.051)^***^ | 0.027 (0.068) | 0.029 (0.026) |
| Woman occupation: Sales and services | 0.076 (0.024)^**^ | 0.065 (0.023)^**^ | 0.038 (0.019)^*^ |
| Woman occupation: (Un)Skilled manual | 0.037 (0.026) |  | 0.013 (0.018) |
| Woman occupation: No work or household chores | 0.047 (0.027) | 0.081 (0.025)^**^ | -0.054 (0.019)^**^ |
| Partner occupation: Professional/technical/managerial | -0.021 (0.027) | -0.017 (0.037) | 0.049 (0.020)^*^ |
| Partner occupation: Sales and services | 0.040 (0.024) | 0.023 (0.019) | 0.050 (0.021)^*^ |
| Partner occupation: (Un)Skilled manual | 0.013 (0.018) |  | 0.021 (0.015) |
| Partner occupation: No work or household chores | 0.072 (0.037) | -0.015 (0.042) | 0.077 (0.034)^*^ |
| Wealth index: Lowest | -0.049 (0.033) | -0.122 (0.044)^**^ | -0.081 (0.030)^**^ |
| Wealth index: Second | -0.078 (0.031)^*^ | -0.131 (0.040)^**^ | -0.133 (0.028)^***^ |
| Wealth index: Middle | -0.087 (0.032)^**^ | -0.157 (0.039)^***^ | -0.115 (0.026)^***^ |
| Wealth index: Fourth | -0.073 (0.028)^**^ | -0.119 (0.039)^**^ | -0.088 (0.024)^***^ |
| Household possesses radio/TV: Yes | 0.028 (0.014) | -0.011 (0.018) | -0.016 (0.013) |
| Residence: Urban | 0.043 (0.030) | 0.063 (0.035) | 0.043 (0.022) |
| Distance is a problem: Yes | -0.035 (0.014)^**^ | -0.045 (0.015)^**^ | -0.046 (0.013)^***^ |
| Birth order: 3rd - 4th | -0.047 (0.020)^*^ | -0.079 (0.024)^**^ | -0.050 (0.017)^**^ |
| Birth order: 5th + | -0.079 (0.024)^**^ | -0.105 (0.031)^***^ | -0.052 (0.022)^*^ |
| Gestation age at first visit: 4 - 6 months | -0.070 (0.018)^***^ | -0.049 (0.020)^*^ | -0.020 (0.013) |
| Gestation age at first visit: 7+ months | -0.125 (0.021)^***^ | -0.085 (0.030)^**^ | -0.079 (0.026)^**^ |
| ANC visits: 0 visits | -0.172 (0.023)^***^ | -0.204 (0.038)^***^ | -0.293 (0.040)^***^ |
| R squared | 0.121 | 0.107 | 0.075 |
| Observations | 4947 | 4868 | 10263 |

Standard errors in parentheses ^*^ *p* < 0.05, ^**^ *p* < 0.01, ^***^ *p* < 0.001

**Supplementary Table 4: Predictors of utilisation of ANC4+ visits and Skilled Birth Attendance combined**

|  | 2006 | 2011 | 2016 |
| --- | --- | --- | --- |
| Woman age at last birth | 0.016 (0.007)^*^ | -0.002 (0.009) | 0.009 (0.007) |
| Woman age at last birth squared | -0.000 (0.000) | 0.000 (0.000) | -0.000 (0.000) |
| Woman years of schooling | 0.013 (0.003)^***^ | 0.007 (0.003)^**^ | 0.010 (0.002)^***^ |
| Partner years of schooling | 0.001 (0.001) | 0.000 (0.001) | 0.000 (0.001) |
| Household size | -0.001 (0.003) | 0.002 (0.003) | 0.003 (0.002) |
| Woman occupation: Professional/technical/managerial | 0.147 (0.051)^**^ | 0.130 (0.047)^**^ | 0.007 (0.023) |
| Woman occupation: Sales and services | 0.059 (0.028)^*^ | 0.061 (0.022)^**^ | 0.059 (0.019)^**^ |
| Woman occupation: (Un)Skilled manual | 0.044 (0.036) |  | -0.017 (0.017) |
| Woman occupation: No work or household chores | 0.052 (0.030) | 0.055 (0.021)^*^ | -0.013 (0.018) |
| Partner occupation: Professional/technical/managerial | 0.012 (0.027) | 0.008 (0.032) | 0.053 (0.019)^**^ |
| Partner occupation: Sales and services | 0.062 (0.020)^**^ | 0.020 (0.020) | 0.028 (0.018) |
| Partner occupation: (Un)Skilled manual | 0.040 (0.019)^*^ |  | 0.052 (0.013)^***^ |
| Partner occupation: No work or household chores | 0.042 (0.035) | -0.067 (0.040) | 0.027 (0.029) |
| Wealth index: Lowest | -0.164 (0.036)^***^ | -0.200 (0.041)^***^ | -0.094 (0.026)^***^ |
| Wealth index: Second | -0.152 (0.034)^***^ | -0.154 (0.034)^***^ | -0.088 (0.024)^***^ |
| Wealth index: Middle | -0.161 (0.031)^***^ | -0.139 (0.033)^***^ | -0.076 (0.022)^***^ |
| Wealth index: Fourth | -0.137 (0.031)^***^ | -0.108 (0.031)^***^ | -0.039 (0.022) |
| Household possesses radio/TV: Yes | 0.004 (0.016) | 0.006 (0.016) | 0.008 (0.012) |
| Residence: Urban | 0.054 (0.034) | 0.035 (0.029) | 0.027 (0.018) |
| Distance is a problem: Yes | -0.028 (0.012)^*^ | -0.014 (0.014) | -0.029 (0.012)^*^ |
| Birth order: 3rd - 4^th^ | -0.068 (0.021)^**^ | -0.043 (0.021)^*^ | -0.057 (0.016)^***^ |
| Birth order: 5th + | -0.063 (0.028)^*^ | -0.049 (0.028) | -0.074 (0.022)^***^ |
| Gestation age at first visit: 4 - 6 months | -0.134 (0.018)^***^ | -0.173 (0.021)^***^ | -0.226 (0.012)^***^ |
| Gestation age at first visit: 7+ months | -0.311 (0.021)^***^ | -0.378 (0.031)^***^ | -0.539 (0.019)^***^ |
| R squared | 0.187 | 0.156 | 0.153 |
| Observations | 4947 | 4868 | 10263 |

Standard errors in parentheses ^*^ *p* < 0.05, ^**^ *p* < 0.01, ^***^ *p* < 0.001

**Supplementary Table 5: Predictors of utilisation of ANC4+ visits, Skilled Birth Attendance and PNC combined**

|  | 2006 | 2011 | 2016 |
| --- | --- | --- | --- |
| Woman age at last birth | 0.016 (0.006)^***^ | 0.005 (0.008) | 0.012 (0.006)^*^ |
| Woman age at last birth squared | -0.000 (0.000)^**^ | -0.000 (0.000) | -0.000 (0.000) |
| Woman years of schooling | 0.008 (0.002)^***^ | 0.010 (0.002)^***^ | 0.012 (0.002)^***^ |
| Partner years of schooling | 0.001 (0.000)^**^ | 0.000 (0.001) | 0.000 (0.001) |
| Woman occupation: Professional/technical/managerial | 0.208 (0.051)^***^ | 0.097 (0.054)^*^ | 0.014 (0.024) |
| Woman occupation: Sales and services | 0.081 (0.023)^***^ | 0.042 (0.018)^**^ | 0.049 (0.020)^**^ |
| Woman occupation: (Un)Skilled manual | 0.013 (0.023) |  | -0.013 (0.016) |
| Woman occupation: No work or household chores | 0.031 (0.023) | 0.040 (0.018)^**^ | -0.054 (0.017)^***^ |
| Partner occupation: Professional/technical/managerial | -0.005 (0.023) | -0.033 (0.031) | 0.048 (0.020)^**^ |
| Partner occupation: Sales and services | 0.037 (0.020)^*^ | 0.024 (0.017) | 0.035 (0.018)^*^ |
| Partner occupation: (Un)Skilled manual | 0.000 (0.013) |  | 0.042 (0.013)^***^ |
| Partner occupation: No work or household chores | 0.059 (0.031)^*^ | -0.037 (0.034) | 0.058 (0.026)^**^ |
| Wealth index: Lowest | -0.063 (0.026)^**^ | -0.135 (0.034)^***^ | -0.096 (0.026)^***^ |
| Wealth index: Second | -0.067 (0.025)^***^ | -0.135 (0.032)^***^ | -0.116 (0.025)^***^ |
| Wealth index: Middle | -0.081 (0.024)^***^ | -0.132 (0.032)^***^ | -0.120 (0.022)^***^ |
| Wealth index: Fourth | -0.067 (0.024)^***^ | -0.105 (0.029)^***^ | -0.079 (0.024)^***^ |
| Household possesses radio/TV: Yes | 0.018 (0.010)^*^ | -0.007 (0.012) | 0.001 (0.012) |
| Residence: Urban | 0.002 (0.025) | 0.047 (0.028)^*^ | 0.016 (0.019) |
| Distance is a problem: Yes | -0.016 (0.010)^*^ | -0.019 (0.012) | -0.023 (0.012)^**^ |
| Birth order: 3rd - 4^th^ | -0.055 (0.016)^***^ | -0.054 (0.020)^***^ | -0.058 (0.016)^***^ |
| Birth order: 5th + | -0.068 (0.021)^***^ | -0.062 (0.025)^**^ | -0.060 (0.021)^***^ |
| Gestation age at first visit: 4 - 6 months | -0.098 (0.017)^***^ | -0.116 (0.020)^***^ | -0.130 (0.012)^***^ |
| Gestation age at first visit: 7+ months | -0.176 (0.018)^***^ | -0.233 (0.023)^***^ | -0.333 (0.020)^***^ |
| R squared | 0.134 | 0.128 | 0.104 |
| Observations | 4947 | 4868 | 10263 |

Standard errors in parentheses ^*^ *p* < 0.10, ^**^ *p* < 0.05, ^***^ *p* < 0.01
